# Environmental Risk Assessment of Potential Toxic Elements in Co-Pyrolysis of Sludges and Plastics Based on Machine Learning

**DOI:** 10.3390/toxics14040289

**Published:** 2026-03-28

**Authors:** Jialing Liu, Xingyu Feng, Xiyu Zhao, Sen Yang, Liyang Dong, Asani Oneka Green, Xu Wang, Qing Huang

**Affiliations:** Key Laboratory of Agro-Forestry Environmental Processes and Ecological Regulation of Hainan Province/Hainan Provincial Academician Team Innovation Center/International Joint Research Center for the Control and Prevention of Environmental Pollution on Tropical Islands of Hainan Province/School of Environment Science and Engineering/School of Computer Science and Technology, Hainan University, Haikou 570228, China; 18932299069@163.com (J.L.);

**Keywords:** sludge–plastic co-pyrolysis, potential toxic elements, machine learning, environmental risk assessment

## Abstract

Co-pyrolysis of sludge and plastics has gradually emerged as a crucial technical approach for waste reduction and resource recovery. This study develops high-precision, interpretable prediction models and quantifies the contributions of core risk factors to environmental risks. Based on the experimental datasets from 2015 to 2025, which include operational parameters and eight potential toxic elements (PTEs) with four chemical speciation fractions: acid-soluble/exchangeable (F1), reducible (F2), oxidizable (F3), and residual (F4), we constructed six machine learning models. Based on the experimental datasets from 2015 to 2025, which include operational parameters and eight potential toxic elements (PTEs) chemical speciation (F1–F4), we constructed six machine learning models. Feature importance analysis and Shapley Additive Explanation (SHAP) analysis were employed to identify core risk factors and interpret the model’s decision logic. Results indicate that XGBoost, Random Forest and CatBoost outperform other models, achieving test accuracies of 0.94, 0.92, and 0.90, with weighted F1-Scores of 0.94, 0.92, and 0.90, respectively. Feature importance highlights the most important features for the six different models, with Cd-F4, As-F1, and Cu-F4 contributing most significantly to the model predictions. SHAP analysis quantified the contributions of each feature to the model predictions, verified Cd-F4 as the primary risk discriminant, and further revealed that F1 and F4 of PTEs are key factors in distinguishing risk levels. This study proposes an interpretable machine learning framework, providing a theoretical basis for the optimization of the sludge and plastic co-pyrolysis process and the assessment of potential risks.

## 1. Introduction

With the public concern over environmental quality and human health increasing [1], stabilization and solidification, detoxification, resources and reduction pollutants of municipal solid waste are the key topics in environmental science [2]. The environmental safety associated with these processes has garnered considerable attention [3].

Sludge production has been steadily increasing in volume in recent years; the dry sludge output from wastewater treatment plants in prefecture-level and above cities reached 11.63 million tons in 2020 [4], and is expected to reach 170.6 million tons by 2036 [5]. Therefore, achieving low-cost resource utilization and harmless treatment of sludge has become a focal point of the current research. Traditional sludge disposal methods mainly include landfilling, incineration, and land application [6]. However, under certain conditions, these methods may pose secondary pollution risks through leachate generation [7], gas emissions, and long-term accumulation. In contrast, sludge pyrolysis technology offers advantages in rapid volume reduction, harmlessness, and resource recovery, and has become one of the mainstream technologies for sludge disposal [8].

Meanwhile, due to their light weight, durability, low cost, and ease of processing, plastics have been widely used in industrial production [9], daily life, healthcare, and agricultural production across various sectors [10]. Global plastic waste is projected to hit 460 million tons annually in 2030, and the unrecycled fraction will be incinerated [11], landfilled, or released to the environment. It is projected that this figure will increase to 12 billion tons by 2050 [12]. The growth in plastic production and consumption poses significant environmental challenges [13]. Previous studies have shown that sludge has a relatively high ash content and a significantly lower organic matter proportion compared to biomass [14], resulting in its low calorific value and insufficient energy supply during pyrolysis treatment [15]. As a polymer waste, waste plastics exhibit characteristics of high calorific value and low ash content [16], which are highly compatible with the requirements of sludge pyrolysis—it can not only supplement energy for sludge pyrolysis to achieve system energy balance, but also improves the performance indicators of pyrolytic char while reducing disposal costs [17], thereby enhancing the technical feasibility of this process. This holds significant implications for the proper disposal of sludge [18].

However, during the co-pyrolysis of sludge and plastics, the presence of plastics may alter the volatilization behavior and chemical speciation of potential toxic elements (PTEs) by changing the reaction atmosphere [19], ash composition, and mineral structure. This complicates the environmental behavior of PTEs within the system [20].

The potential environmental risks of PTEs are not only related to their total concentrations, but are also closely linked to their distribution across various chemical forms [21]. It is generally accepted that exchangeable and reducible forms of PTEs exhibit higher mobility and bioavailability [22], whereas residual forms tend to be more stable. However, in the co-pyrolysis system of sludge and plastics, various operational parameters [23]—such as reaction temperature, residence time, plastic blending ratio, heating rate, and treatment atmosphere—often change simultaneously [24], leading to nonlinear and multifactorial interactions between the speciation of PTEs and environmental risk. Consequently, single-factor risk assessments are insufficient to comprehensively reflect the actual situation. Current research primarily focuses on energy conversion and process optimization in the co-pyrolysis of sludge and plastics, while studies on the potential ecological and environmental risks associated with this process are relatively limited [25]. However, this aspect is crucial for the proper disposal of sludge and plastics through co-pyrolysis and warrants further in-depth investigation. In light of this, the development of an environmental risk assessment model that integrates multiple operational parameters and PTEs chemical speciation characteristics is essential for identifying the potential environmental risk of PTEs under varying treatment conditions. Recently, machine learning methods have been increasingly applied in environmental pollution analysis and risk assessment [26], owing to their ability to process high-dimensional, multivariable, and nonlinear data, offering new insights for identifying environmental risk in complex systems [27].

Based on the research background outlined above, this study systematically compiles experimental data from the published literature to construct a dataset containing operational parameters and various PTEs’ chemical speciation characteristics. Machine learning techniques are then applied to develop a classification model to predict the environmental risk levels of PTEs during the co-pyrolysis of sludge and plastics. Feature importance analysis and interpretability methods are used to examine the effects of different factors on variations in environmental risk. This study aims to provide quantitative references for the identification of PTEs’ environmental risks and the environmental management of sludge co-pyrolysis under complex operating conditions.

## 2. Methodology

### 2.1. Data Collection

The experimental data utilized in this study were obtained from the published literature focusing on PTEs speciation in sludge co-pyrolysis with plastics. Among these sludges, sewage sludge constitutes the majority of the samples, while textile sludge and dyeing sludge account for a negligible proportion. The dataset encompassed a comprehensive set of parameters characterizing the thermal treatment process and resulting PTEs distributions. The compiled dataset contains 162 individual experimental records, collected from peer-reviewed journal articles published between 2015 and 2025; the search keywords for the relevant articles include sludge, plastics, co-pyrolysis, and potential environmental risks. Twenty high-quality articles were selected from the published literature, from which 162 data points were extracted.

These records include measurements of five primary operational parameters and speciation distributions of eight PTEs across four chemical fractions.

The operational parameters comprised the reaction temperature (ranging from ambient to 950 °C), reaction time (0–240 min), plastic addition ratio (0–100%), heating rate (0–40 °C/min) and pyrolysis atmosphere (N_2_, CO_2_/O_2_, AIR and N_2_/O_2_). Seven plastic types were investigated: polyvinyl chloride (PVC), polyethylene (PE), polypropylene (PP), polystyrene (PS), polyethylene terephthalate (PET), polyamide 6 (PA6), and other polymer combinations. PTEs speciation data were recorded for eight elements—cadmium (Cd), chromium (Cr), manganese (Mn), nickel (Ni), lead (Pb), zinc (Zn), arsenic (As), and copper (Cu)—each characterized by four fractions (F1–F4) based on the European Community Bureau of Reference (BCR) sequential extraction procedure. The F1 fraction represents the acid-soluble/exchangeable form, F2 corresponds to the reducible form, F3 indicates the oxidizable form, and F4 represents the residual fraction.

The operational parameters and speciation of PTE characteristics of the waste co-pyrolysis system offer valuable insights into the transformation behavior and environmental risk of PTEs under different thermal treatment conditions. The wide temperature range (0–950 °C) reflects various thermal treatment scenarios, from ambient storage to high-temperature incineration, while reaction times spanning 0–240 min capture both short-term and extended exposure effects. The plastic addition ratios (0–100%) enable systematic evaluation of the plastic waste’s influence on the speciation of PTE evolution during co-pyrolysis processes.

### 2.2. Data Preprocessing

Data preprocessing involves several critical steps to prepare the dataset for model development and analysis. The preprocessing workflow consisted of three main stages: categorical variable encoding, missing value imputation, and data standardization.

#### 2.2.1. Categorical Variable Encoding

For the categorical variable “plastic type” label encoding was applied to transform textual categories into numerical representations that are suitable for machine learning algorithms. A total of seven distinct plastic types were identified in the dataset, including PVC, PE, PP, PS, PET, PA6 and other polymer combinations. The encoding was performed using scikit-learn’s LabelEncoder, which assigned unique integer values (0–7) to each category while maintaining their distinctiveness for subsequent analysis.

#### 2.2.2. Missing Value Imputation

Due to the heterogeneous nature of the literature-compiled data, certain experimental records contained missing values for specific features. To ensure data completeness and preserve the underlying relationships among variables, an advanced iterative imputation strategy was employed. The IterativeImputer algorithm, based on the multiple imputation by chained equations (MICE) framework, was utilized with a Random Forest Regressor as the base estimator. This approach leverages the non-linear modeling capability of Random Forests to capture complex inter-feature dependencies during the imputation process [28].

The IterativeImputer algorithm, based on the MICE framework, was employed with a Random Forest Regressor (containing 10 decision trees) as the conditional estimator for each feature. The imputation process iterates all features with missing values in a round-robin fashion; in each round, a feature’s missing values are predicted using a Random Forest trained on the observed values of that feature, with all remaining features as inputs. This cycle was repeated for a maximum of 15 rounds to ensure the convergence of the imputed values.

This method iteratively estimates each missing value by modeling it as a function of other features, thereby maintaining the multivariate structure of the data and minimizing information loss compared to simple statistical imputation methods. For the categorical variable (plastic type), the imputed numerical codes were rounded to the nearest integer and decoded back to their original textual labels using the inverse transform of the LabelEncoder, ensuring semantic consistency throughout the dataset. This two-stage encoding–imputation–decoding workflow preserved both the categorical nature of plastic types and the integrity of the imputation process.

#### 2.2.3. Data Standardization

To ensure comparability across features with different scales and units, all numerical variables were standardized using Z-score normalization. The standardized values were calculated as follows:

(1) $$\begin{array}{c} X_{std}=\frac{X_{raw}-\mu }{\sigma } \end{array}$$

Here, *X_std_* represents the standardized value, *X_raw_* denotes the original value, *μ* indicates the mean of each feature, and *σ* represents the standard deviation of each feature. This transformation achieved a uniform scale for all variables (mean = 0, standard deviation = 1) and approximated a normal distribution, facilitating effective model training and preventing features with larger numerical ranges from dominating the learning process.

The preprocessed dataset, comprising 162 complete records with 38 features (five operational parameters, one categorical identifier for plastic type, and 32 speciations of PTEs percentages), was subsequently employed for model development and feature importance analysis.

### 2.3. ML Model Development

Machine learning (ML) techniques simulate human learning behavior to address complex classification tasks. In this study, ML models were developed and evaluated to predict PTEs’ environmental risk levels in waste co-pyrolysis systems. The selected models included one tree-based model—Decision Tree (DT)—and five ensemble models—Random Forest (RF), Extreme Gradient Boosting (XGBoost), Light Gradient Boosting Machine (LightGBM), Gradient Boosting Decision Tree (GBDT), and CatBoost. These models have demonstrated strong applicability in environmental risk assessment and classification scenarios [29].

The individual ecological risk (*E_r_*) is calculated as follows:

(2) $$\begin{array}{c} E_{r}=T_{r\;}\times C_{f} \end{array}$$

(3) $$RI=\sum E_{r}$$

where *T_r_* is the Hakanson toxicity response coefficient (Cd = 30, As = 10, Pb = 5, Ni = 5, Cu = 5, Cr = 2, Zn = 1, Mn = 1), and *C_f_* is derived from the ratio of bioavailable PTEs fractions (F1 + F2 + F3) to the residual fraction (F4) [30]. The comprehensive Risk Index (RI) is the sum of all individual *E_r_* values. Four risk categories are defined by dividing RI values based on the actual data distribution characteristics of the compiled dataset and in combination with the classification criteria of the existing relevant studies on PTEs’ ecological risk in sludge–plastic co-pyrolysis systems: low (RI ≤ 50), moderate (50 < RI ≤ 150), high (150 < RI ≤ 300), and considerable (RI > 300) [31].

The dataset was randomly split into training and testing sets, with 70% allocated for training and 30% reserved for testing. Stratified sampling (stratify = y) was applied to preserve proportional class representation in both subsets. To enhance prediction reliability, five-fold stratified cross-validation was applied during the training process. Specifically, the original training dataset was partitioned into five subsets while maintaining the class distribution proportions; each subset served as the validation set once, while the remaining subsets were used for training. This process was repeated five times, and the average performance across iterations was used as the evaluation metric during hyperparameter optimization. Learning curves demonstrating model convergence as a function of the training set size are presented in Figure S2.

All model development was implemented in the Python 3.9 environment, using open-source libraries such as Scikit-learn 1.8.0, XGBoost 2.1.2, LightGBM 4.5.0, and CatBoost 1.2.7, with GBDT, Random Forest, and Decision Tree implemented via Scikit-learn 1.8.0. The model performance was assessed using four key statistical metrics: accuracy, precision, recall, and F1-Score. Accuracy measures the overall proportion of correctly classified samples, reflecting the model’s general classification capability. Precision evaluates the proportion of true positive predictions among all positive predictions, indicating the model’s ability to avoid false positives. Recall quantifies the proportion of true positive predictions among all actual positive samples, representing the model’s sensitivity to identifying positive cases. F1-Score provides a harmonic mean of precision and recall, offering a balanced evaluation metric that is particularly valuable for imbalanced datasets.

The calculation formulas for these metrics are as follows:

(4) $$\begin{array}{c} Accuracy=\frac{TP+TN}{TP+TN+FP+FN} \end{array}$$

(5) $$\mathrm{Precision}=\frac{TP}{TP+FP}$$

(6) $$\begin{array}{c} Recall=\frac{TP}{TP+FN} \end{array}$$

(7) $$\begin{array}{c} F1\text{-}Score=2\times \frac{\mathrm{Precision}\times \mathrm{Recall}}{\mathrm{Precision}+\mathrm{Recall}} \end{array}$$

Here, *TP* represents true positives, *TN* denotes true negatives, *FP* indicates false positives, and *FN* represents false negatives. For multi-class classification, the weighted averages were calculated based on the support (number of true instances) for each class. These metrics collectively provide a comprehensive evaluation of classification accuracy and model performance.

### 2.4. Model Optimization and Interpretation

To optimize model performance, Bayesian optimization was employed to determine the optimal hyperparameters for each model, using the Optuna 4.8.0 framework.

Bayesian optimization is a sequential model-based optimization technique that builds a probabilistic model of the objective function and uses it to select the most promising hyperparameters to evaluate. Specifically, the Optuna Tree-structured Parzen Estimator (TPE) sampler was used to sequentially propose candidate hyperparameter configurations. For each candidate, the model was trained and evaluated via 5-fold stratified cross-validation on the training set, and the weighted F1-Score averaged across folds served as the optimization objective to be maximized. This approach is more efficient than a grid search or random search, particularly for expensive objective functions. For each model, 30 optimization trials were conducted to explore the hyperparameter space, with the weighted F1-Score as the optimization objective.

The hyperparameter search spaces for each model are summarized in Table 1. The ranges were defined based on prior knowledge and computational constraints to balance the model complexity and generalization capability. After optimization, the best-performing configuration for each model was identified and used for final model training and evaluation.

Following hyperparameter optimization, the best-performing models were retrained on the entire training set (70% of data) and evaluated on the independent test set (30% of data). To analyze the contribution of input features to risk level predictions, feature importance analysis was conducted for each model. Tree-based models (RF, XGBoost, LightGBM, GBDT, CatBoost, and DT) inherently provide feature importance scores based on the reduction in impurity or gain contributed by each feature during tree construction.

Subsequently, the Shapley Additive Explanation (SHAP) method was employed to provide model-agnostic interpretability and quantify the marginal contribution of each feature to individual predictions. The SHAP values, derived from cooperative game theory [32], offer a unified measure of feature importance that satisfies desirable properties such as local accuracy, missingness, and consistency.

The SHAP framework decomposes each prediction into the sum of feature contributions, enabling both global feature importance ranking and local explanation of individual predictions. This comprehensive analysis not only improved the model accuracy but also deepened the understanding of complex relationships between the operational conditions, speciation of PTEs, and environmental risk levels.

## 3. Results and Discussion

### 3.1. Operational Parameters’ Data Characteristics and Distribution

**Figure 1:**
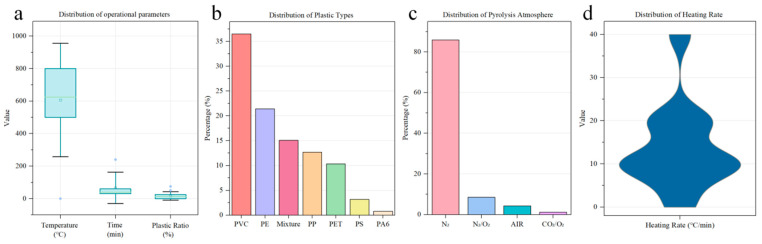
Data characteristics distribution in co-pyrolysis systems. (**a**) Distribution of operational parameters, including temperature, reaction time, and plastic addition ratio; (**b**) frequency distribution of plastic types in the dataset; (**c**) frequency distribution of pyrolysis atmosphere in the dataset; and (**d**) distribution of heating rate in the dataset.

The operational parameters of the sludge and plastic co-pyrolysis system offer valuable insights under different thermal treatment conditions; the data characteristics revealed several key patterns that are critical for understanding the speciation of PTEs’ evolution during co-pyrolysis processes (Figure 1).

#### 3.1.1. Distribution Characteristics of Operating Parameters

The temperature distribution exhibits a wide range, from ambient conditions to 950 °C, with a median value around 600 °C, reflecting diverse thermal treatment scenarios from low-temperature pyrolysis to high-temperature incineration (Figure 1a).

A median reaction time of approximately 60 min is observed, encompassing a wide span of treatment periods from immediate exposure to extended durations reaching 240 min (Figure 1b).

Nitrogen (N_2_) is the most frequently used atmosphere, accounting for over 85.89% of the experiments (Figure 1c). This preference is likely due to nitrogen’s inert properties, which help to prevent oxidation during thermal treatment, ensuring a more controlled pyrolysis process [33]. Mixed N_2_/O_2_ atmospheres only account for a small proportion (around 8.59%), which is typically used to study the combined effects of both reducing and oxidizing conditions on PTEs migration and transformation during co-pyrolysis [34]. The use of CO_2_/O_2_ and air atmospheres is significantly less common, comprising approximately 1.23% and 4.29% of the dataset, respectively. These atmospheres can promote the oxidation of metals and facilitate their transformation into more mobile forms, thus influencing the speciation of PTEs and environmental risk. The distribution pattern suggests that most of the experiments are designed to simulate controlled pyrolysis environments, while a smaller subset investigates reactive atmospheres to assess specific treatment effects. The majority of experiments were conducted at heating rates between 0 and 20 °C/min, with the most common heating rate being 10 °C/min, accounting for approximately 47.06% of the dataset (Figure 1d), reflecting standard thermal treatment conditions for sludge and plastics. This range ensures a balanced decomposition of organic materials while minimizing the volatilization of PTEs. A smaller proportion of experiments employed higher heating rates, with values ranging from 20 to 40 °C/min, accounting for approximately 35.29% of the dataset. The relatively wide distribution of heating rates indicates that the dataset encompasses a broad spectrum of experimental conditions, which may affect the transformation behavior of PTEs and their subsequent environmental risks.

#### 3.1.2. Distribution Characteristics of Plastic Factors

In this dataset, the plastic addition ratio demonstrates a right-skewed distribution, with most experiments conducted at relatively low plastic ratios (median ~10%), although some studies investigated high co-pyrolysis ratios up to 100%.

The distribution of plastic types reveal that polyvinyl chloride (PVC) dominates the dataset, with over 36.51% (Figure 1b). Differences in the chemical composition of plastics may influence the speciation and volatilization behavior of PTEs during thermal treatment [31,35]. For chlorine-containing plastic, hydrogen chloride (HCl) released during pyrolysis can react with PTEs to form more mobile chloride species [36], which may increase the proportions of the F1 and F2 fractions. Consequently, such plastics tend to exert a more pronounced influence on the speciation of PTEs [37]. Non-chlorinated plastics such as PE, PP, and PS have a weaker interference on the speciation of PTEs [38] and are more likely to promote the enrichment of PTEs into stable forms (F4). This distribution pattern suggests that the dataset encompasses a comprehensive range of operational conditions that are representative of practical co-pyrolysis scenarios.

### 3.2. Model Performance Comparison and Evaluation Results

**Figure 2:**
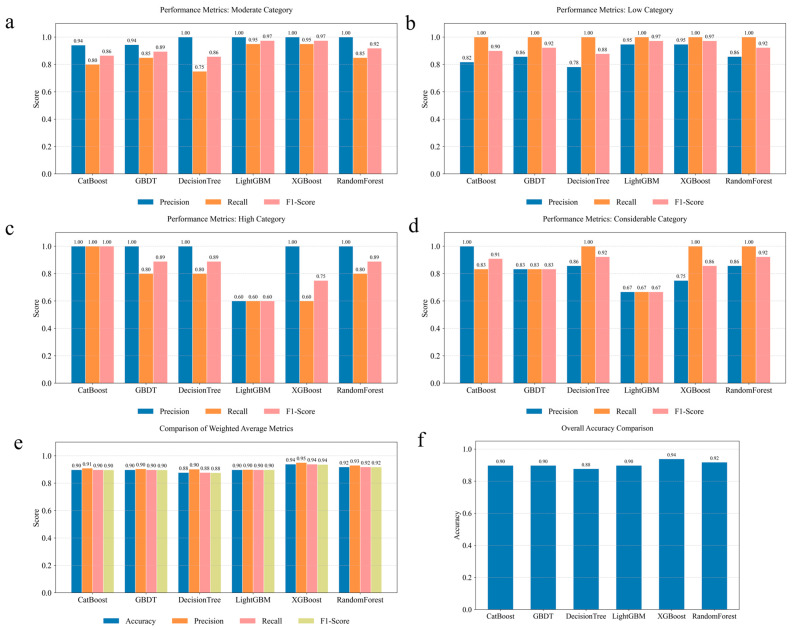
Performance comparison of six machine learning models across different risk categories. (**a**) Moderate-risk categories; (**b**) low-risk categories; (**c**) high-risk categories; (**d**) considerable-risk categories; (**e**) weighted average performance metrics across all categories; and (**f**) overall accuracy comparison for all models. Top four panels show detailed performance metrics (precision, recall, F1-Score).

Six machine learning models were developed and evaluated for their prediction performance in environmental risk level classification. For the moderate-risk category (Figure 2a), XGBoost and Random Forest achieved perfect precision (1.00), while all models maintained high F1-Scores ranging from 0.86 to 0.97. This superior performance across most models for the moderate category suggests that samples in this risk level possess distinct feature patterns that facilitate accurate classification.

The low-risk category exhibited excellent model performance across all algorithms, with five models (CatBoost, GBDT, XGBoost, Random Forest, and LightGBM) achieving F1-Scores between 0.92 and 0.97 (Figure 2b). Decision Tree demonstrated a strong but slightly lower performance, with F1-Scores of 0.88. The consistently high recall values (0.90–1.00) across all models indicate their robust capability to correctly identify low-risk samples, minimizing false negatives, which is crucial for environmental risk assessment to avoid underestimating potential hazards.

For the high-risk category (Figure 2c), CatBoost achieved perfect classification (1.00 for all metrics), demonstrating exceptional performance for this category. GBDT, Decision Tree, and Random Forest maintained strong performance, indicating partial misclassification of high-risk samples. XGBoost showed an F1-Score of 0.75, with a Recall of 0.60, suggesting that more high-risk samples were misclassified as other categories. LightGBM exhibited a comparatively lower performance for this category, with precision, recall, and F1-Score all at 0.60, suggesting challenges in capturing the specific feature patterns that are characteristic of high-risk samples.

The considerable risk category presented the greatest classification challenge for most models (Figure 2d). Decision Tree and Random Forest achieved perfect recall (1.00), with F1-Scores of 0.92, demonstrating a strong capability to distinguish this severe risk level. XGBoost maintained a robust performance, with an F1-Score of 0.86. However, CatBoost, GBDT, and LightGBM showed reduced performance, with F1-Scores of 0.91, 0.83, and 0.67, respectively, indicating potential limitations in capturing the complex feature interactions that are characteristic of considerable-risk conditions.

The weighted average performance metrics (Figure 2e) and overall accuracy (Figure 2f) provide a comprehensive evaluation across all risk categories. XGBoost, Random Forest and CatBoost outperform other models, achieving test accuracies of 0.94, 0.92, and 0.90, with weighted F1-Scores of 0.94, 0.92, and 0.90, respectively (Figure 2e). Among the top-performing models, XGBoost showed the highest prediction accuracy, and was therefore selected as the best model for subsequent SHAP analysis (Figure 2f). This advantage is consistent with the results reported in relevant ensemble model studies, further confirming the strong capability of tree-based ensemble algorithms in capturing nonlinear features [39].

**Figure 3:**
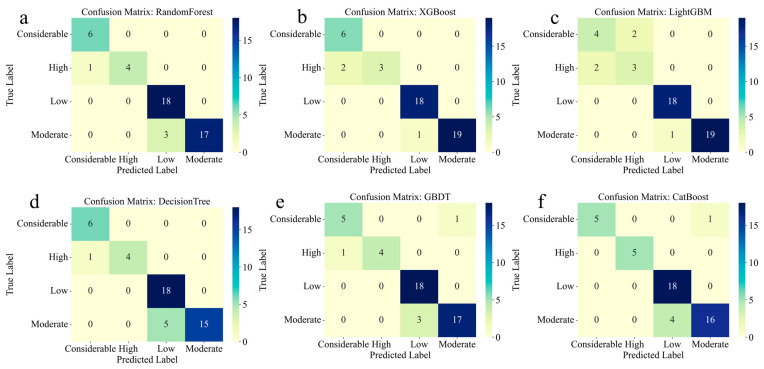
Confusion matrices for six machine learning models. (**a**) Random Forest; (**b**) XGBoost; (**c**) LightGBM; (**d**) Decision Tree; (**e**) GBDT; and (**f**) CatBoost. The matrices show the distribution of true labels versus predicted labels across four risk categories: considerable, high, low, and moderate. Darker colors indicate higher frequencies of classification.

Random Forest and Decision Tree exhibit identical confusion patterns, achieving perfect classification for the considerable (6/6) and low (18/18) risk categories, with minor misclassifications in the high category (four correct, one misclassified as considerable) (Figure 3a,d). The moderate category shows 17 correct classifications, with three misclassified as low, demonstrating the model’s slight tendency to underestimate risk in this category.

XGBoost demonstrates strong overall performance but shows more dispersed errors than Random Forest (Figure 3b). While achieving perfect low-risk classification (18/18) and strong moderate performance (19/20), it misclassifies two high-risk samples as considerable and two considerable samples as high, indicating occasional confusion between the two highest risk categories. This pattern suggests that XGBoost may face challenges in distinguishing extreme risk conditions, though its overall accuracy remains high.

LightGBM exhibits the most significant classification challenges among the ensemble models (Figure 3c). While maintaining perfect low risk classification (18/18) and strong moderate performance (19/20), it shows considerable confusion in the high category, with two samples misclassified as considerable and two misclassified as the considerable category. Additionally, four considerable samples are misclassified as high, indicating a systematic difficulty in discriminating between these two categories.

GBDT and CatBoost demonstrate nearly identical confusion patterns and represent the most robust classification performance among all models (Figure 3e,f). Both achieve perfect or near-perfect classification across all risk categories. GBDT shows only minor errors, with one considerable sample misclassified as moderate and three moderate samples misclassified as low, while CatBoost exhibits a similar pattern, with one considerable and four moderate samples misclassified. The perfect classification of low-risk samples (18/18) and strong discrimination of high-risk samples (four to five correct out of five) demonstrate their exceptional capability to handle a multi-class environmental risk assessment.

Collectively, these results underscore the superior effectiveness of ensemble tree-based models, particularly CatBoost, GBDT, and Random Forest, in capturing complex relationships between operational parameters, speciation of PTEs, and environmental risk levels. The consistent high performance across multiple risk categories and the clear patterns in confusion matrices demonstrate the reliability of these models for practical environmental risk assessment applications. The success of these models provides a robust foundation for future applications in PTE risk prediction and waste co-pyrolysis process optimization.

### 3.3. Feature Importance and Model Interpretation

#### 3.3.1. Feature Importance Analysis

Using tree-based machine learning models, the top 10 most important features for six different models are highlighted, revealing both consensus and model-specific patterns in feature contribution.

**Figure 4:**
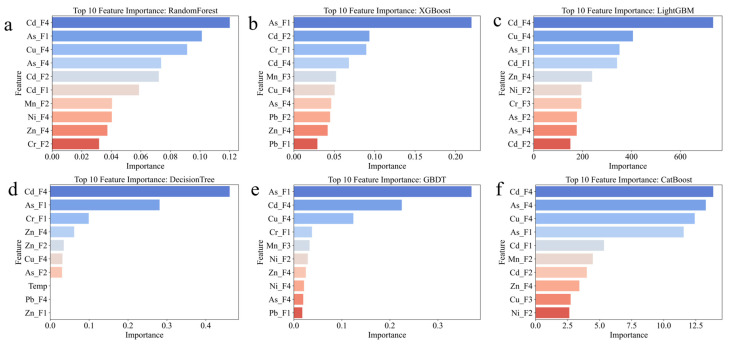
Top 10 feature importance analysis for six machine learning models. (**a**) Random Forest, (**b**) XGBoost; (**c**) LightGBM; (**d**) Decision Tree; (**e**) GBDT; and (**f**) CatBoost. Features are displayed on the y-axis, with importance scores on the x-axis. Colors range from blue (higher importance) to red (lower importance) within each model’s top 10 features.

Random Forest analysis identified Cd-F4 (cadmium residual fraction) as the most important feature, with an importance score of 0.12, followed by As-F1 (arsenic exchangeable fraction) and Cu-F4 (copper residual fraction) (Figure 4a). The prominence of Cd-F4 suggests that the stability of cadmium in its most immobile form serves as a key indicator of the overall environmental risk. The content of Cd-F4 exhibits a direct correlation with the environmental risk, and when its proportion lies within an appropriate range, the corresponding environmental risk tends to be relatively low. This is consistent with the nonlinear correlation captured by the model; thus, its stability serves as a key indicator for characterizing the overall environmental risk [40].

The high ranking of As-F1 indicates that bioavailable arsenic plays a significant role in risk assessment, as the F1 fraction represents the most easily mobilized and potentially toxic form. Other important features include As-F4, Cd-F2, Cd-F1, Mn-F2, Ni-F4, Zn-F4, and Cr-F2, demonstrating that multiple PTEs and their various fractions contribute to risk determination, with cadmium-related features appearing three times in the top 10.

XGBoost analysis presents a different prioritization pattern, ranking As-F1 as the most critical feature with an importance score exceeding 0.20, nearly double that of the second-ranked feature Cd-F2 (Figure 4b). As-F1 is prone to volatilization or leaching during co-pyrolysis [41], making it an important indicator of environmental risk.

This emphasizes the dominant role of exchangeable arsenic in XGBoost’s risk classification logic. The prominence of Cr-F1 (chromium exchangeable fraction) as the third most important feature is noteworthy, as chromium typically exists predominantly in stable forms, suggesting that even small amounts of mobile chromium significantly influence risk assessment. The following features include Cd-F4, Mn-F3, Cu-F4, As-F4, Pb-F2, Zn-F4, and Pb-F1, indicating that a diverse set of PTE fractions contribute to the model’s decision-making process.

LightGBM analysis identified Cd-F4 as overwhelmingly dominant, with an importance score exceeding 600, far surpassing all other features (Figure 4c). This exceptional prominence suggests that LightGBM relies heavily on the cadmium residual fraction as the primary discriminator for risk classification. Cu-F4, As-F1, and Cd-F1 follow as secondary important features, though with substantially lower importance scores. The remaining top 10 features include Zn-F4, Ni-F2, Cr-F3, As-F2, As-F4, and Cd-F2. This hierarchical pattern with one dominant feature may explain LightGBM’s comparatively lower performance in distinguishing between the high and considerable risk categories, as observed in the confusion matrix analysis.

Decision Tree analysis showed a more balanced feature importance distribution, with Cd-F4 leading at approximately 0.40 importance, followed by As-F1 and Cr-F1 at much lower but comparable levels (Figure 4d). The presence of operational parameters such as Temp (temperature) in the top 10 features distinguishes Decision Tree from other models, highlighting the importance of thermal treatment conditions in risk determination. Other important features include Zn-F4, Zn-F2, Cu-F4, As-F2, Pb-F4, and Zn-F1, demonstrating a focus on the speciation of zinc, with three zinc-related features in the top 10.

GBDT analysis identified As-F1 as the most important feature, consistent with XGBoost’s pattern, followed by Cd-F4 and Cu-F4 (Figure 4e). The subsequent features include Cr-F1, Mn-F3, Ni-F2, Zn-F4, Ni-F4, As-F4, and Pb-F1, showing a diverse representation of multiple PTEs. The ranking pattern suggests that GBDT balances the consideration of both highly mobile fractions (F1) and stable residual fractions (F4) across different metals, potentially contributing to its superior overall classification performance.

CatBoost analysis revealed Cd-F4 as the most important feature, followed by As-F4, Cu-F4, and As-F1 (Figure 4f). The prominence of residual fractions (F4) for three different metals (Cd, As, Cu) in the top four positions indicates CatBoost’s emphasis on overall PTEs stability as a primary risk indicator. The subsequent features include Cd-F1, Mn-F2, Cd-F2, Zn-F4, Cu-F3, and Ni-F2, demonstrating comprehensive consideration of multiple speciation forms across various PTEs.

A cross-model comparison reveals several consistent patterns. Cadmium-related features, particularly Cd-F4 and Cd-F1, appear in the top 10 for all six models, confirming cadmium as a universal key indicator of environmental risk in waste co-pyrolysis systems. The arsenic exchangeable fraction (As-F1) consistently ranks highly across models, appearing in all six top 10 lists, emphasizing the critical importance of bioavailable arsenic in risk assessment. The copper residual fraction (Cu-F4) appears in five out of six models’ top 10 features, suggesting that copper stability plays a significant role in overall risk determination. The convergence of multiple models on these key features strengthens confidence in their practical significance for environmental management.

However, notable differences exist in feature prioritization strategies. XGBoost and GBDT show a preference for exchangeable fractions (F1), while LightGBM and CatBoost emphasize residual fractions (F4), suggesting that different internal algorithms capture distinct aspects of PTEs’ behavior. The relative balance or imbalance of feature importance distributions correlates with model performance, as models with more balanced importance distributions (XGBoost, Random Forest and CatBoost) generally achieved superior classification accuracy across all risk categories. These insights provide valuable guidance for feature selection in future modeling efforts and highlight the critical PTE fractions that should be prioritized in experimental monitoring and risk management strategies.

#### 3.3.2. SHAP-Based Model Interpretation

**Figure 5:**
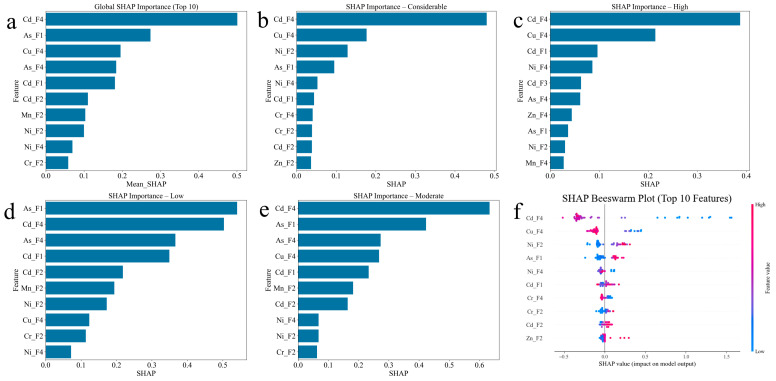
SHAP feature importance analysis across global and category-specific perspectives. (**a**) Global SHAP importance for top 10 features, showing mean absolute SHAP values; (**b**) SHAP importance for considerable-risk category; (**c**) SHAP importance for high-risk category; (**d**) SHAP importance for low-risk category; (**e**) SHAP importance for moderate-risk category; and (**f**) SHAP beeswarm plot illustrating the relationship between feature values and SHAP contributions for top 10 features. In the beeswarm plot, each point represents a sample, with color indicating feature value (red = high, blue = low) and x-position showing SHAP impact on model output.

The SHAP analysis was performed on the XGBoost. In the SHAP (Shapley Additive Explanation) analysis, Cd-F4 showed the highest mean absolute SHAP value (approximately 0.5), confirming its dominant role as the primary risk discriminator identified in the model-based feature importance analysis (Figure 5a). As-F1, Cu-F4, As-F4, Cd-F1, Cd-F2, Mn-F2, Ni-F2, Ni-F4, and Cr-F2 follow in descending order, demonstrating strong consistency with the tree-based feature importance rankings while providing additional quantitative evidence of their contributions to risk classification. This alignment is further consistent with the well-established finding that the residual (F4) and exchangeable (F1) fractions of PTEs serve as key drivers of environmental risk in thermochemical treatment systems [42].

For the considerable risk category, Cd-F4 remains dominant with SHAP values exceeding 0.4, followed by Cu-F4, Ni-F2, and As-F1, indicating that a high residual cadmium fraction combined with specific copper, nickel, and arsenic patterns characterizes this highest risk level (Figure 5b). The high-risk category shows a similar top three features (Cd-F4, Cu-F4, Cd-F1), but with different relative contributions, suggesting that subtle variations in the speciation of PTEs profiles distinguish high from considerable risk (Figure 5c). The low-risk category exhibits As-F1 as the most important feature, followed closely by Cd-F4 and As-F4, emphasizing that low bioavailable arsenic combined with specific cadmium patterns serves as a key indicator of a minimal environmental hazard (Figure 5d). The moderate-risk category demonstrates a more balanced importance distribution among Cd-F4, As-F1, As-F4, Cu-F4, and Cd-F1, reflecting the intermediate nature of this risk level, where no single feature dominates the classification decision (Figure 5e).

Focusing on the top 10 features, the SHAP beeswarm plot delivers a holistic understanding of feature importance and how feature values directionally affect model predictions. Each point represents a sample, with the x-axis showing the SHAP value (impact on model output) and color indicating feature value (red = high, blue = low). Cd-F4 shows wide SHAP value distribution with predominantly positive contributions from high values (red points on the right), confirming that elevated cadmium residual fractions generally increase the predicted risk, though the relationship exhibits notable variability across samples. Cu-F4 demonstrates a more complex pattern with both positive and negative SHAP values across different feature value ranges, explaining its context-dependent influence observed in the waterfall plot analysis. As-F1 shows a clear trend where high values (red) concentrate in positive SHAP regions, indicating that increased bioavailable arsenic consistently elevates risk predictions. The beeswarm plot reveals that multiple features exhibit non-linear relationships with risk classification, as evidenced by the curved distributions of points, validating the choice of tree-based ensemble models over linear approaches for this environmental risk-assessment task.

**Figure 6:**
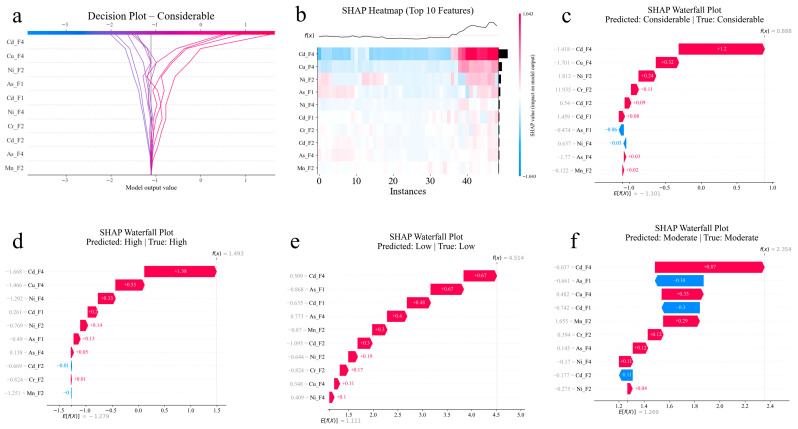
SHAP-based model interpretation for environmental risk classification. (**a**) Decision plot for considerable-risk category, showing cumulative feature contributions across samples; (**b**) SHAP heatmap displaying feature contributions for top 10 features across multiple samples; (**c**–**f**) SHAP waterfall plots illustrating individual feature contributions for correctly predicted samples from four risk categories: (**c**) considerable risk (f(x) = 0.888); (**d**) high risk (f(x) = 1.493); (**e**) low risk (f(x) = 4.514); and (**f**) moderate risk (f(x) = 2.354). Red bars indicate positive contributions (increasing predicted risk), while blue bars represent negative contributions (decreasing predicted risk).

Insights into the role of specific feature combinations in driving classification decisions at the sample level are derived from detailed SHAP analysis results for individual predictions across different risk categories (Figure 6). With lines representing individual samples converging toward the final prediction value, the features are ordered by their average absolute SHAP contribution, with Cd-F4, Cu-F4, Ni-F2, As-F1, Cd-F1, Ni-F4, Cr-F2, Cd-F2, As-F4, and Mn-F2 appearing as the most influential. The convergence pattern reveals that samples classified as considerable risk typically exhibit strong positive contributions from specific cadmium, copper, and nickel features, while samples diverging toward lower risk predictions show negative contributions from these same features. The color gradient from purple to red indicates the progression of prediction values, with red lines representing samples that are strongly predicted as a considerable risk.

The SHAP heatmap presents a compact visualization of feature contributions across the top 10 most important features for a subset of samples (Figure 6b). Each row represents a feature, and each column represents an individual sample. The color intensity indicates the magnitude and direction of the SHAP values, with red representing positive contributions (increasing risk prediction) and blue representing negative contributions (decreasing risk prediction). The f(x) curve at the top shows the final model prediction values for each sample. The heatmap reveals several key patterns: Cd-F4 and Cu-F4 show predominantly strong positive contributions (red regions) across samples with high predicted risk, while features like Ni-F2 and As-F1 exhibit more varied contributions, suggesting their context-dependent influence. The vertical banding pattern in the heatmap indicates that samples cluster into distinct groups based on their feature value combinations, corresponding to different risk categories. The black markers on the right indicate feature values (high or low), providing additional context for interpretation.

The SHAP waterfall plot starts from a base value (E[f(X)] = 0.888), representing the average model output, and sequentially adds the contribution of each feature to reach the final prediction: f(x) = 0.888, for this specific sample (Figure 6c). Cd-F4 provides the largest positive contribution (+1.2), strongly pushing the prediction toward considerable risk. Cu-F4 contributes an additional +0.32, further reinforcing the high risk classification. Ni-F2 adds +0.24, continuing the upward trend. Interestingly, Cr-F2 shows a negative contribution (−0.11), slightly reducing the risk prediction, but this is outweighed by positive contributions from Cd-F2 (+0.09) and Cd-F1 (+0.08). The remaining features (As-F1, Ni-F4, As-F4, Mn-F2) contribute small adjustments, collectively fine-tuning the final prediction. This waterfall visualization clearly demonstrates that for this considerable-risk sample, the dominant factors are high values of Cd-F4 and Cu-F4, while other features provide secondary modulation.

For a correctly classified high-risk sample, the SHAP waterfall plot uncovers a different feature contribution pattern, where the sample attains a final prediction of f(x) = 1.493 from the base value (E[f(X)] = 1.493) through a specific set of feature impacts (Figure 6d). Cd-F4 again provides the largest contribution (+1.38), confirming its central role across multiple risk categories. However, Cu-F4 shows a moderate negative contribution (−0.55), contrasting with its positive contribution in the considerable-risk sample. This suggests that lower copper residual fraction values characterize high risk compared to considerable risk. Ni-F4 provides a positive contribution (+0.33), while Cd-F1 adds +0.2. The remaining features (Ni-F2, As-F1, As-F4, Cd-F2, Cr-F2, Mn-F2) contribute smaller adjustments. The distinct feature contribution pattern illustrates how the model differentiates between the high- and considerable-risk categories through subtle variations in the speciation of PTEs’ profiles.

The SHAP waterfall plot of a correctly predicted low-risk sample demonstrates the model’s approach to identifying low environmental risk conditions (Figure 6e). Starting from a substantially different base value (E[f(X)] = 4.514), this sample reaches a final prediction of f(x) = 4.514 through predominantly negative contributions from key features. Cd-F4 provides the largest negative contribution (−0.67), indicating that for this low-risk sample, the cadmium residual fraction has a lower value compared to higher risk samples, paradoxically reducing the predicted risk because the model has learned that extreme stability (very high F4) and extreme instability (very low F4) can both indicate elevated risk, depending on context with other features. As-F1 shows a large negative contribution (−0.67), suggesting low bioavailable arsenic characterizes this low-risk condition. Cd-F1 adds −0.48, further confirming the reduced cadmium mobility. The other features (As-F4, Mn-F2, Cd-F2, Ni-F2, Cu-F4, Ni-F4, Cr-F2) show varied contributions, collectively maintaining the prediction in the low-risk range. Synchronized decreases in the exchangeable fractions of multi-element systems are a reliable indicator of a low environmental hazard in thermochemical treatment processes [38]. This pattern reveals that low-risk conditions are characterized by coordinated reductions in multiple bioavailable fractions (F1) across different PTEs.

The SHAP waterfall plot of a correctly predicted low-risk sample demonstrates the model’s approach to identifying low environmental risk conditions (Figure 6e).

For the moderate-risk sample, the plot starts from E[f(X)] = 2.354, and the sample reaches f(x) = 2.354 through a balanced combination of positive and negative feature contributions. Cd-F4 provides a strong negative contribution (−0.87), pushing the prediction downward from considerable/high towards moderate (Figure 6f). As-F1 shows a large negative contribution (−0.38), which is consistent with the pattern observed in low-risk samples, but to a lesser extent. Conversely, Cu-F4 and Cd-F1 provide substantial positive contributions (+0.33 and +0.3, respectively), moderating the downward trend and maintaining the prediction in the moderate-risk range. Mn-F2, Cr-F2, As-F4, Ni-F4, Cd-F2, and Ni-F2 contribute smaller adjustments. This balanced pattern of counteracting contributions reflects the intermediate nature of moderate-risk conditions, where neither extreme stability nor extreme mobility dominates, but rather a nuanced combination of multiple PTEs fractions determines the overall risk level.

By comparing SHAP analyses across the four risk categories, several important insights emerge. First, Cd-F4 consistently appears as the most influential feature across all risk levels, but its contribution direction and magnitude vary substantially, confirming its role as a primary risk discriminator. Second, the interplay between residual fractions (F4) and exchangeable fractions (F1) determines risk categorization, with high and considerable risks characterized by specific combinations that differ from low and moderate risks. Third, individual samples within the same risk category can exhibit different feature contribution patterns, highlighting the model’s ability to capture multiple pathways to the same risk classification. Finally, the consistency between SHAP analysis and model-based feature importance rankings strengthens confidence in the identified key features, providing a robust, multi-dimensional perspective for understanding factors influencing the environmental risk in waste co-pyrolysis systems (Figure 4). This interpretability framework enhances the model’s practical applicability for environmental management decision-making and provides actionable insights for risk mitigation strategies.

## 4. Conclusions

This study presents an interpretable machine learning framework to assess environmental risks in sludge–plastic co-pyrolysis, addressing the limitations of traditional chemical analysis in predicting variable operational conditions. Among the six evaluated models, Random Forest, XGBoost, and CatBoost show superior classification performance. Feature importance and SHAP analysis identify Cd-F4, As-F1, and Cu-F4 as top contributors, revealing that the interplay between residual fractions (F4) and exchangeable fractions (F1) of PTEs is a key mechanism distinguishing risk categories. This work provides a theoretical basis and technical support for co-pyrolysis parameter optimization, risk prevention, and engineering applications. After recalibration to regional waste stream characteristics, the model can support real-time risk warning, intelligent process regulation, and quantitative references for the experimental design and environmental risk control standards.

The limitations include that the BCR sequential extraction method is a static batch process, which is unable to fully reflect the dynamic speciation of PTEs changes during continuous co-pyrolysis, and its extraction efficiency is susceptible to the reagent concentration and temperature, potentially causing slight deviations in speciation quantification and risk classification. Practical application requires local data collection for model recalibration, especially for region-specific waste streams, to enable actionable recommendations for feedstock optimization and process control to reduce environmental risks.

## Figures and Tables

**Table 1:** Hyperparameter search spaces and optimal configurations.

Model	Hyperparameter	Search Range	Optimal Value
Decision Tree	max-depth (Maximum tree depth)	[3, 20]	9
	min-samples-split (Min. samples to split)	[2, 20]	6
	criterion (Split quality measure)	[‘gini’, ‘entropy’]	entropy
Random Forest	n-estimators (Number of trees)	[100, 500]	306
	max-depth (Maximum tree depth)	[3, 15]	14
XGBoost	n-estimators (Number of boosting rounds)	[100, 500]	155
	learning-rate (Boosting learning rate)	[0.01, 0.2] (log scale)	0.0769
	max-depth (Maximum tree depth)	[3, 10]	3
LightGBM	n-estimators (Number of boosting rounds)	[100, 500]	282
	learning-rate (Boosting learning rate)	[0.01, 0.2] (log scale)	0.0518
	num-leaves (Maximum number of leaves)	[20, 150]	80
	max-depth (Maximum tree depth)	[3, 12]	7
GBDT	n-estimators (Number of boosting rounds)	[100, 500]	469
	learning-rate (Boosting learning rate)	[0.01, 0.2] (log scale)	0.1482
	max-depth (Maximum tree depth)	[3, 8]	5
CatBoost	iterations (Number of boosting rounds)	[100, 500]	242
	learning-rate (Boosting learning rate)	[0.01, 0.2] (log scale)	0.0252
	depth (Maximum tree depth)	[3, 10]	5

Note: Log scale indicates logarithmic sampling in the specified range. The optimal values were determined through Bayesian optimization with 30 trials per model using stratified 5-fold cross-validation.

## Data Availability

The original contributions presented in this study are included in the Supplementary Material. Further inquiries can be directed to the corresponding author.

## Supplementary Materials

The following supporting information can be downloaded at: https://www.mdpi.com/article/10.3390/toxics14040289/s1, Figure S1. Overall accuracy and risk-specific classification performance of six machine learning models. Figure S2. Learning curves for six machine learning models using 5-fold stratified cross-validation. Figure S3. Bayesian hyperparameter optimization using Optuna (30 trials per model). Table S1. Raw unprocessed dataset. Table S2. Sample Distribution of Comprehensive Potential Ecological Risk Index (RI) Levels. Table S3. Highly correlated feature pairs (|r| > 0.7).

## Abbreviations

The following abbreviations are used in this manuscript:

BCR	European Community Bureau of Reference
