# Supplementary material for: Environmental Risk Assessment of Potential Toxic Elements in Co-Pyrolysis of Sludges and Plastics Based on Machine Learning
Source: Toxics. 2026 Mar 28;14(4):289. doi: 10.3390/toxics14040289 (PMC13120067; doi:10.3390/toxics14040289)
Supplement: Supplementary file 1 [file toxics-14-00289-s001.zip › Supplementary Material (Figure S1-S3 Tbale S2-S3).pdf]

Supplementary Material

Environmental Risk Assessment of Potential Toxic Elements in Co-pyrolysis of Sludges and Plastics Based on Machine Learning

Jialing Liu[1], Xingyu Feng[1], Xiyu Zhao[1], Sen Yang[1], Liyang Dong[1],Asani Oneka Green[1], Xu Wang[1], Qing Huang[1*]

[a] Key Laboratory of Agro-Forestry Environmental Processes and Ecological Regulation of Hainan Province / Hainan Provincial Academician Team Innovation Center / International Joint Research Center for the Control and Prevention of Environmental Pollution on Tropical Islands of Hainan Province / School of Environment Science and Engineering / School of Computer Science and Technology, Hainan University, Haikou 570228, China

* Corresponding Author.

Email: huangqing@hainanu.edu.cn (Qing Huang)

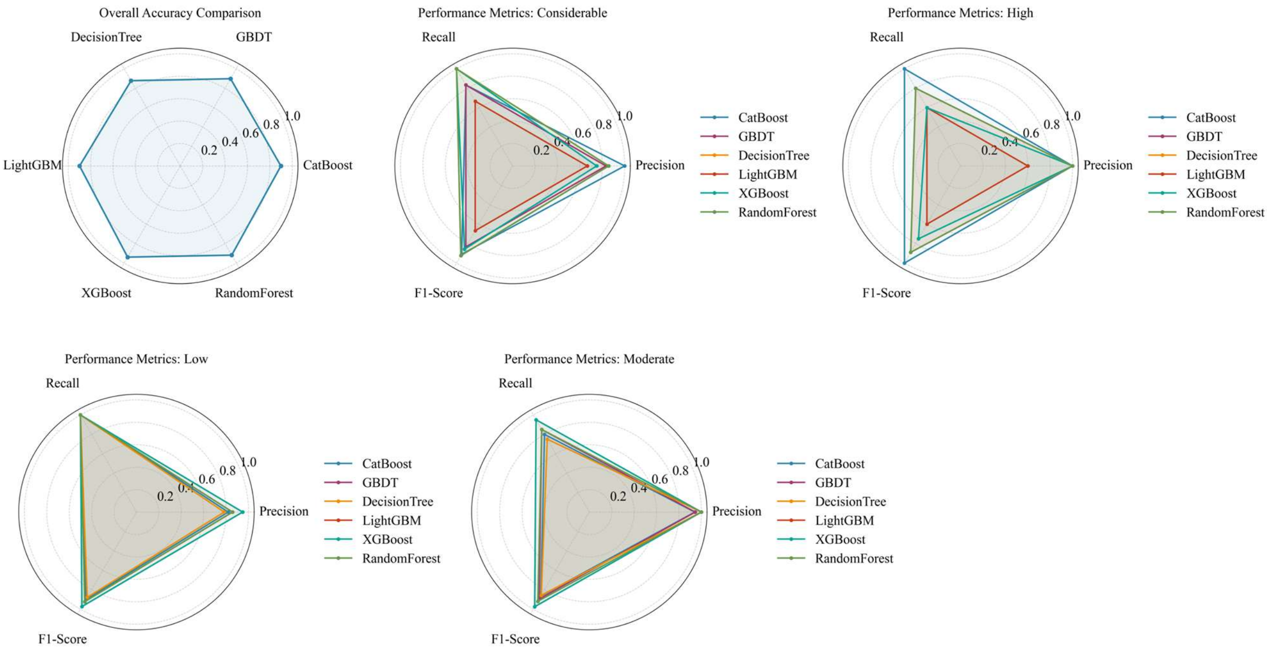

Figure S1. Overall accuracy and risk-specific classification performance of six machine learning models.

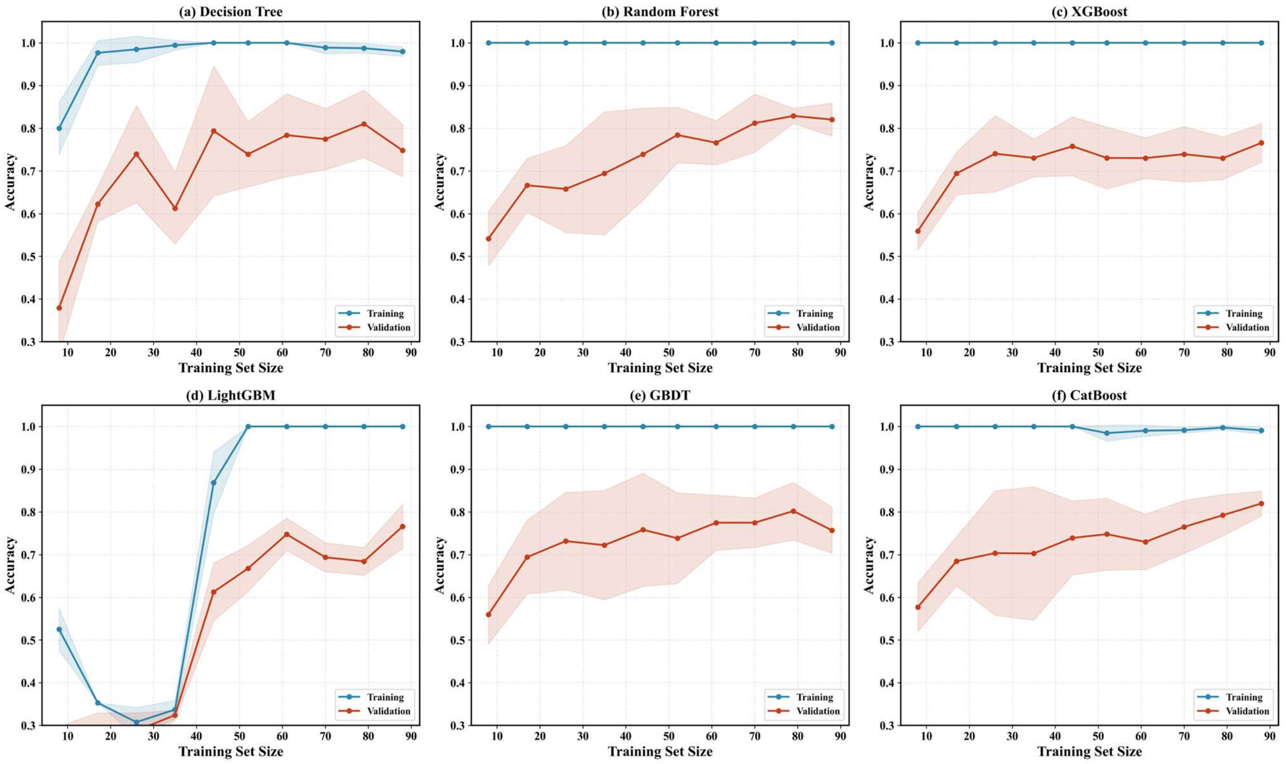

Figure S2. Learning curves for six machine learning models using 5-fold stratified cross-validation. The shaded regions represent ±1 standard deviation.

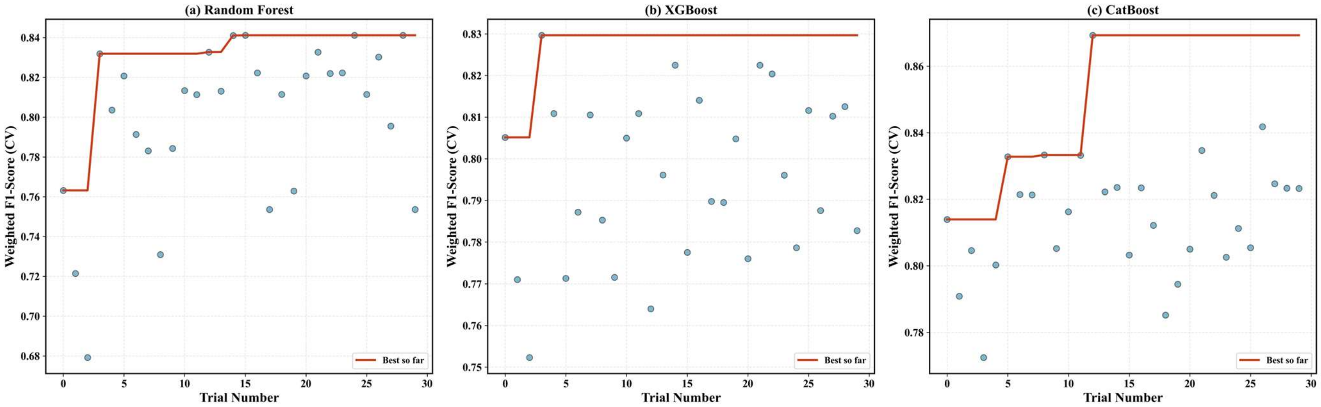

Figure S3. Bayesian hyperparameter optimization using Optuna (30 trials per model). Each point represents a trial; the red line tracks the best F1-score achieved so far.

Table S1. Raw unprocessed dataset

Table S2. Sample Distribution of Comprehensive Potential Ecological Risk Index (RI) Levels

Risk Level	Threshold Range	Sample Size
Low	RI≤50	7 samples
Moderate	50＜RI≤150	13 samples
High	150＜RI≤300	32 samples
Considerable	RI＞300	110 samples

Table S3. Highly correlated feature pairs (|r| > 0.7).

Feature 1	Feature 2	Pearsonr
Ni_F3	Ni_F4	−0.846
Cr_F3	Cr_F4	−0.831
Mn_F4	Ni_F4	0.829
As_F1	As_F4	−0.826
Pb_F2	Pb_F4	−0.807
Cd_F3	Cd_F4	−0.784
Zn_F3	Zn_F4	−0.734
Zn_F1	Cu_F1	0.729
Zn_F3	Cu_F3	0.726
Mn_F3	Ni_F4	−0.725
Mn_F3	Ni_F3	0.717
Cd_F2	Ni_F2	0.701
